# Types of Fever

**Published:** 1887-12

**Authors:** 


### TYPES OF FEVER.

The prevailing type of fever during the last few months throughout the South and in many sections of the North has been *typho-malarial.* In many portions of Northern Georgia it has been unusually prevalent, occuring even in dry, elevated regions, while heretofore it was almost unknown It is certainly an interesting and important subject of inquiry as to the cause of its presence in these healthful places during the last year.

Many physicians refuse to acknowledge that we have a special type of fever to which the name of *typho-malarial* can be appropriately applied, but we have seen much of the disease in this very healthful region of country, and we are satisfied of its mixed character, and that the name typho-malarial is highly applicable, and well expressess its peculiar character. We have seen it commence in all respects as an ordinary intermittent, with no marked indication at the start of the continued or typhoid form ; and yet it would be found that it was not at all amenable to the ordinary treatment with quinine—for days the physician would push his quinine in increasing doses, the paroxysms continuing, the intermissions growing less marked, the tongue becoming pointed and red, with a constant headache and often, though not always, a tendency to diarrhœa, forcing upon him the conviction that a typhoid element was therein combined with the ordinary malarial characteristics.

Our experience in the treatment of a dozen or more of these cases in the months of July, August and September last, was that they are undoubtedly mixed cases, evincing both typhoid and malarial features; that quinine does not control then, and in large doses is a positive injury; that the septinary law existed in every instance, the disease terminating only on the 7th, 14th, 21st and sometimes running through a number of these periods. In one of these cases only did the typhoid element assume complete control as indicated by the meteorism, the sordes and the peculiar diarrhœa, and the low muttering delirium; and this typhoid state was developed only after the third week, in which there appeared to be a relapse and a change to a fully marked typhoid condition. The treatment found best was the expectant. No remedy was found, after many experiments, to arrest the fever, and the patient did best under a mild course of treatment directed to his comfort, and to guarding against congestions or local inflammations, and thus carefully conducting him through the inevitable and self-limited phases of the di ease.

The temperature rated from 101 or 102 in the morning to 103 to 104 in the afternoon. During the pyrexia we had the patient frequently sponged with tepid or cool water, and administered every hour about ¼ of a gtt. of aconite with 2 to 3 gtts. of gelsemium. Sometimes large wet compresses were applied to the chest and abdomen, often with the effect of letting down the temperature and giving quiet to the patient. Opiates were frequently given at night when there was unusual restlessness; mustard was applied to any point where pain existed, and so every symptom was treated as it arose. Quinine was given regularly in the morning hours, sooner or later according to the particular period at which the daily increase of fever occurred, but without any special effect as could be perceived. It was hoped that its tonic effect would support the patient in some measure, and that it might assert its antiperiodic power and thus arrest the malady. Arsenic was tried in a few instances without marked results, and so the salycilate of ammonium and other agents. In one case gelsemium was pushed to its full constitutional effect without arresting the disease. The diet given was usually milk in moderate quantities; butter milk was found best adapted to a majority of cases, and the plan so often urged of administering large quantities of sweet milk with a view to supporting the patient was found injurious and often wholly impracticable, the patient not being able to retain it upon the stomach.

